# Childhood tuberculosis treatment outcome and its association with HIV co-infection in Ethiopia: a systematic review and meta-analysis

**DOI:** 10.1186/s41182-020-00195-x

**Published:** 2020-02-18

**Authors:** Getaneh Mulualem Belay, Chalachew Adugna Wubneh

**Affiliations:** Department of Pediatrics and Child Health Nursing, School of Nursing, College of Medicine and Health Sciences, University of Gondar, Gondar, Ethiopia

**Keywords:** Childhood, Ethiopia, HIV co-infection, Treatment outcome, Tuberculosis

## Abstract

**Background:**

Tuberculosis is the second leading cause of death from an infectious disease worldwide, next to HIV. Hence, initiating and determining the national tuberculosis treatment program and outcome is crucial. However, the childhood tuberculosis treatment outcome in Ethiopia was not investigated.

**Objective:**

This study determined the pooled estimate of childhood tuberculosis treatment outcome and its association with HIV co-infection.

**Methods:**

PubMed, Google Scholar, Web of Science, reference lists of included studies, and Ethiopian institutional research repositories were used to retrieve all available studies. Searching was limited to the studies that had been conducted in Ethiopia and published in the English language. In this study, observational studies, including cohort, cross-sectional, and case-control studies, were included. The estimate of childhood tuberculosis treatment outcome was determined using a weighted inverse variance random-effects model. The overall variation between studies was checked by the heterogeneity test (*I*^2^). The Joanna Briggs Institute (JBI) quality appraisal criteria were used for quality assessment of the studies. The summary estimates were presented with forest plots and tables. Publication bias was also checked with the funnel plot and Egger’s regression test. The outcome measures were successful and unsuccessful treatment outcomes. Successful treatment outcomes are defined as patients who are cured and treatment completed, whereas, an unsuccessful treatment outcome means those patients with defaulter, failure, and death treatment outcomes.

**Result:**

To estimate the overall pooled estimate of successful treatment outcome, 6 studies with 5389 participants were considered. Consequently, the overall pooled estimate of successful treatment outcome was 79.62% (95% CI 73.22, 86.02) of which 72.44% was treatment completed. On the other hand, unsuccessful treatment outcomes, including treatment failure, defaulter, and death, were 0.15%, 5.36%, and 3.54%, respectively. Moreover, this study found that HIV co-infection was significantly associated with childhood tuberculosis treatment outcomes. Poor treatment outcome was higher among children with HIV co-infection with an odds ratio of 3.15 (95% CI 1.67, 5.94) compared to that of HIV-negative children.

**Conclusion:**

The summary estimate of successful childhood tuberculosis treatment outcome was low compared to the threshold suggested by the World Health Organization. HIV co-infection is significantly associated with poor treatment outcome of childhood tuberculosis. Therefore, special attention is better to be given to children infected with HIV. Moreover, adherence to anti-TB has to be strengthened.

**Trial registration:**

The protocol has been registered in PROSPERO with a registration number of CRD42018110570.

## Background

World Health Organization (WHO) reported that globally, 10.0 million people developed tuberculosis (TB) disease in 2017 of which 1.0 million were children. According to WHO 2018 TB report in 2017, TB caused an estimated 1.3 million deaths among human immunodeficiency virus (HIV)-negative and 300,000 additional deaths from TB- and HIV-co-infected people [1].

Next to HIV, TB is the second leading cause of death from an infectious disease worldwide. The WHO declared TB as a global public health emergency in 1993 when a large number of deaths occurred due to TB [2]. Globally, TB has been reported to be one of the major causes of death among children [3]. Approximately 1 million children are estimated to be infected by TB worldwide of which 75% occurs in the 22 high-burden countries including Ethiopia [4]. In high-TB burden countries, childhood TB constitutes 20–40% case load [5]. The true burden of childhood TB is underestimated because of the challenge in diagnostic accuracy [6]. Childhood TB is a good indication of the ongoing transmission of TB in the community [2]. Accurate diagnosis and successful treatment of people with TB avert millions of deaths each year (an estimated 54 million over the period 2000–2017), but there are still large and persistent gaps in detection and treatment. The latest treatment outcome data for new cases showed a global treatment success rate of 82% in 2018. This is a reduction from 86% in 2013 and 83% in 2015; in countries where notifications had increased, reporting of treatment outcomes had not kept pace [1].

As Ethiopia is one of the highest TB burden countries in the world, the Federal Ministry of Health of Ethiopia is implementing a TB prevention and control program at all levels of the health facility. According to the Ethiopian National Population-Based Tuberculosis Prevalence Survey conducted in 2011, 7.5/100,000 children were smear positive [7]. In Ethiopia, the TB cure rate was 58% for HIV-positive and 89% for HIV-negative children [8]. Tuberculosis was recognized as a major public health problem in Ethiopia more than half a century ago; since the 1960s, tuberculosis controlling effort has been started in the country [7]. The global community has launched the End TB strategy. Intensified research and innovation are two of the pillars to achieve the End TB strategy. Achieving a ≥ 90% TB treatment success rate is one of the top ten priority indicators for monitoring the implementation of the End TB strategy at global and national levels. The other indicator in the End TB strategy is a reduction in the number of TB deaths. Sustainable Development Goals have been planned to reduce death by 75% in 2025 and 90% by 2030 from 2015 baseline [9].

HIV is considered a fuel factor for the TB epidemic. Studies reported that the risk of TB in children having HIV is very high. TB/HIV co-infection often results in disseminated disease, especially in advanced stages of HIV infection, resulting in poorer survival compared to HIV-negative children [8]. The risk of active TB in HIV co-infected children is related to both CD4 count and more indirectly also to viral load. Conversely, restoration of cellular immunity with anti-retroviral therapy partially reverses the TB susceptibility [10]. In Ethiopia, there is the inconsistency of research findings on childhood TB treatment outcomes. Therefore, this study will estimate the pooled childhood TB treatment outcomes and analyze its association with HIV co-infection.

## Methods

### Protocol registration

The protocol of this study has been accessed through a web address (https://www.crd.york.ac.uk/PROSPERO/#myprospero). Moreover, the protocol registration number is CRD42018110570.

### Reporting

The Preferred Reporting Items for Systematic Review and Meta-Analysis (PRISMA) guideline has been utilized to report the findings of this study (Additional file 1).

### Eligibility criteria

#### Inclusion criteria

Observational studies (cohort, case-control, and cross-sectional) that had been employed in Ethiopia either published or unpublished at any time were included in the study. In addition, we included studies that had been written in the English language. In this study, the study participants were children less than 15 years who had taken tuberculosis treatment.

#### Exclusion criteria

Articles without the full texts were excluded after two email contacts with the primary author of the paper. Moreover, editorials, trials, conference papers, qualitative studies, and review articles were excluded.

### Information sources

Articles were retrieved from PubMed, Google scholar, Web of Science, reference lists of included studies, and institutional research repositories (from University of Gondar and Addis Ababa University). However, searching was limited to the studies that had been conducted in Ethiopia and published in the English language. Both published and unpublished research reports that revealed the childhood TB treatment outcomes and/or its association with HIV co-infection in Ethiopia were included.

### Searching strategy

The following searching terms, including “tuberculosis treatment”, “childhood tuberculosis treatment”, “childhood tuberculosis treatment outcome”, “tuberculosis”, “cured”, “completed”, “treatment completed”, “ relapse”, “ treatment failure”, “died”, “ successful TB treatment outcome”, “ unsuccessful TB treatment outcome”, “childhood tuberculosis treatment”, “outcome of tuberculosis treatment”, “factors”, “risk factors”, “ associated factors”, “predictors”, “ HIV co- infection”, and “Ethiopia”, were systematically searched for both as keyword and MeSH terms. The searching string was developed using the Boolean operators “AND” and “OR”. For instance, the PubMed and Web of Science searching strategy was described in Additional file 2. Moreover, the searching strategy that had been used for Google Scholar was illustrated in Additional file 3. The searching date was until August 23, 2019.

### Study selection

Firstly, all available studies from the electronic databases were retrieved. Second, identified studies were imported to Endnote version 7 (Thomson Reuters, London) citation manager and duplicates were removed carefully. Third, two independent authors (GMB and CAW) screened and assessed the titles and abstracts of the studies followed by reviewing the full texts. The discrepancy between the reviewers had been solved through discussion and consensus.

### Quality assessment

Two authors (GMB and CAW) assessed the quality of included studies independently. The Joana Briggs Institute (JBI) critical appraisal tool has been utilized to critically appraise the quality of the studies. The JBI critical appraisal checklist for cohort and cross-sectional studies was employed (Additional 4). The discrepancies between the authors had been solved through discussing, repeating the procedure, and reaching a consensus.

### Data collection process and extraction

After the development of a data extraction excel spread sheet, the following data items had been extracted from the included studies: first author of the study; study area; region; population; design; sample size; proportion of treatment outcome, including cured, treatment completed, treatment failure, died, and relapse; odds ratio of HIV co-infection; proportion of treatment success; log p; and SE log p. Any disagreement between authors had been solved by discussion.

### Summary measures

Treatment completed is a patient who completed treatment but without evidence of failure or with no record to show that sputum or culture results in the last month of treatment and on at least one previous occasion were negative, either because tests were not done or because results are unavailable, or is a patient with TB who completed treatment without evidence of failure, but with no record of sputum smear or culture results, in the last month of treatment. Cured for PTB is a patient with bacteriologically confirmed pulmonary TB at the beginning of treatment who was smear- or culture-negative in the last month of treatment. Cured for other TB is a patient who completed treatment and considered cured. Treatment failure is a TB patient whose sputum smear or culture is positive at 5 months or later during treatment. Died is a TB patient who died from any cause during the treatment. Successful treatment outcome is if pulmonary tuberculosis patients were cured (i.e., negative smear microscopy at the end of treatment and on at least one previous follow-up test) or completed treatment with resolution of symptoms. Unsuccessful TB treatment outcome is if treatment of pulmonary/extra pulmonary tuberculosis patients resulted in treatment failure (i.e., remaining smear positive after 5 months of treatment), default (i.e., patients who interrupted their treatment for two consecutive months or more after registration), or death.

### Synthesis of results

Before doing meta-analysis, the proportion of treatment outcome and the odds ratio of successful treatment outcome among HIV co-infection patients had been transformed to logarithm in the excel spreadsheet. Then, the excel data was exported to STATA version 11 for further analysis. To determine the pooled effect of TB treatment outcomes, a weighted inverse random effect model was employed. The descriptive data were presented using a table. Besides, the point prevalence of each study as well as the overall prevalence was described using a forest plot graph. The forest plot was interpreted as follows: the horizontal line showed the 95% CI and the black box represented the weight of each study. Moreover, an explanatory data analysis using the *I*^2^ test was conducted to assess the random variations between each primary study. In this study, heterogeneity was interpreted as an *I*^2^ value = 0% no heterogeneity, 25% low, 50% moderate, and 75% high [11]. Based on the above tests, the primary studies included in this meta-analysis exhibited a significant random variation (*I*^2^ = with Eggers regression test *p* value < 0.001), which forced us to use a random-effects meta-analysis model to compute pooled effect. Publication bias was assessed by funnel plot and Egger’s test. Statistically significant publication bias was declared at *p* value less than 0.05.

## Result

### Study selection and screening

**Fig. 1:**
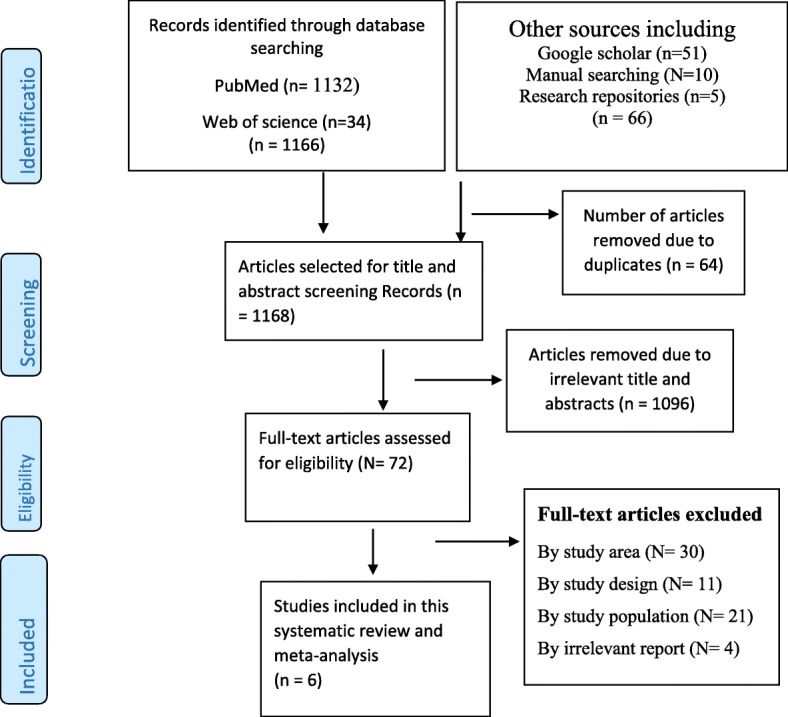
Flow diagram of articles selection and screening

As indicated Fig. 1, totally, we have retrieved 1232 studies which (1132) were from PubMed, (34) Web of Science, (51) Google Scholar, and (10) manual searching of reference lists of the included studies, and the rest (5) were from Ethiopian institutional research repositories. Primarily, 64 research reports were removed due to duplication and 1096 because of irrelevant titles and abstracts. For the full-text review, 72 articles were selected, of which 30 articles were removed due to the study area, 11 due to the study designs, 21 due to the study population, and 4 due to an irrelevant report as to the pre-specified inclusion criteria. Finally, 6 studies were considered in the study.

### Study characteristics

As described in Table 1, a total of six studies with 5389 participants were included in the study. All studies had been published between 2009 and 2017 within a follow-up period of 5 to 10 years. The follow-up period of childhood TB treatment outcomes ranged from 1998 to 2013. Regarding the study area, one study was conducted in the Tigray region [12, 17], two in Addis Ababa [16], one in Amhara [15], one in Oromia [13], and one in SNNPRS [14]. Among the included studies, four [12–14, 16] were conducted with a retrospective cohort study design, and two [15, 17] were retrospective cross-sectional. The minimum and maximum sample sizes were 226 and 2565, respectively. In this review, the highest TB treatment success rate was reported from Tigray and Addis Ababa (85.5%), and the lowest from Oromia (66.4%).

**Table 1:** General characteristics of included studies

Author/year of publication	Study area	Region	Study design	Study population	Follow-up period	Sample size	Treatment success rate	Quality assessment result
Tilahun and Gebre-Selassie/2016 [12]	Addis Ababa Zewuditu Hospital	Addis Ababa	Retrospective cohort	children < 15 years of age	2009–2013	491	85.5	80
Ramos et al./2010 [13]	Arsi zone	Oromia	Retrospective cohort	children < 15 years of age	1998–2007	1029	66.9	75
Muñoz-Sellart et al./2009 [14]	Sidama zone	SNNPRS	Retrospective cohort	children < 15 years of age	2002–2007	851	77	60
Kebede et al./2017 [15]	Gondar	Amhara	Cross-sectional	children < 15 years of age	NA	227	78.9	75
Hailu et al./2014 [16]	Addis Ababa	Addis Ababa	Retrospective cohort	children < 15 years of age	2007–2011	2565	85.5	80
Daemo and Kelbore/2016 [17]	Mekelle	Tigray	Retrospective cross-sectional	children < 15 years of age	2007–2011	226	84	85

### Quality assessment results

Since all included studies were cohort and cross-sectional study design, we assessed the quality of the papers using Joana Briggs Institute (JBI) adapted for cohort and cross-sectional studies. Studies that fitted to 50% and above the quality assessment checklist were considered low risk and included in this study. However, none of the studies were excluded after performing a quality assessment. As indicated in Table 1, the results of quality assessment ranged from 60 to 85%.

### Meta-analysis

**Fig. 2:**
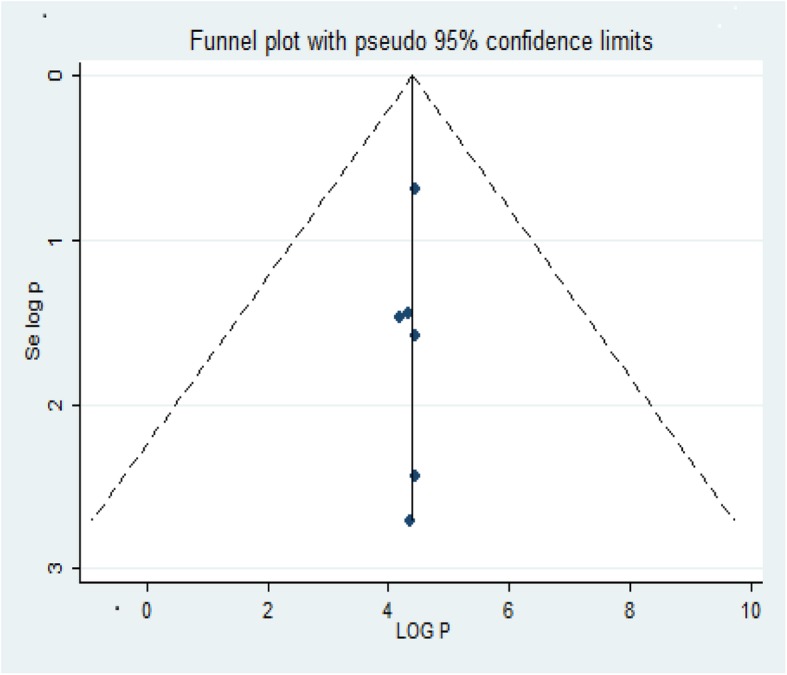
Funnel plot of successful treatment outcome with 95% CI

We performed a funnel plot and Egger’s regression test to determine the publication bias. Hence, the publication bias was not detected as the funnel plot is symmetrical in observation (Fig. 2) and the Egger’s regression test is 0.203.

### Childhood TB treatment outcome

**Fig. 3:**
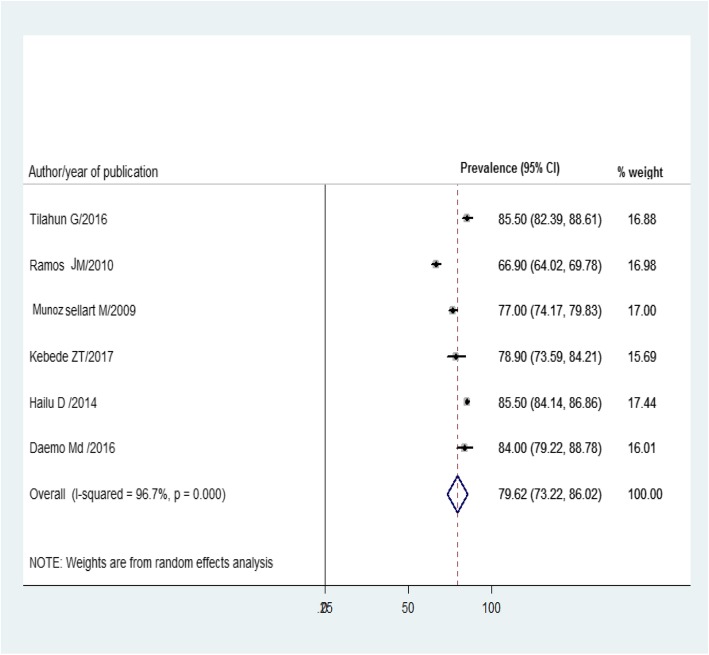
Forest plot of successful childhood TB treatment outcome

**Fig. 4:**
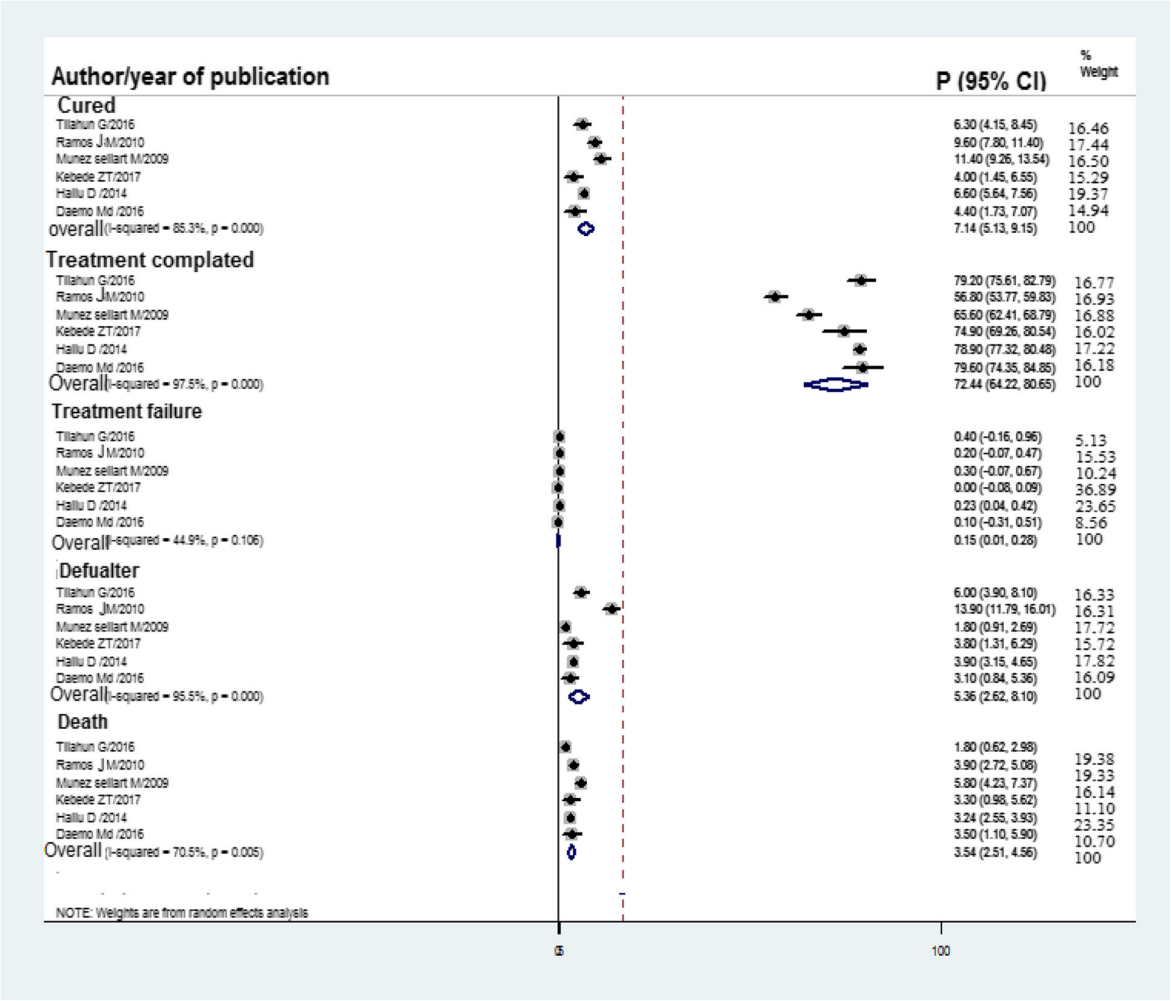
Forest plot of TB treatment outcome

All of the included studies had reported childhood TB treatment outcomes. Consequently, as illustrated in Fig. 3, the overall pooled childhood TB treatment success rate was 79.62% (95% CI 73.22, 86.02) of which 72.44% were treatment completed and 7.1% were cured, whereas unsuccessful TB treatment outcomes were described in Table 2. Moreover, as described in Fig. 4, treatment failure, defaulter, and death were 0.15%, 5.36%, and 3.54%, respectively.

**Table 2:** Childhood tuberculosis treatment outcome

Region	Successful TB treatment outcome		Unsuccessful TB treatment outcome		
	Treatment completed (%)	Cured (%)	Failure (%)	Died (%)	Defaulter (%)
Amhara	74.9	4	0.0	3.3	3.8
Oromia	56.8	9.6	0.2	3.9	13.9
Addis Ababa	78.9	6.6	0.23	3.24	3.9
Tigray	79.4	5.35	0.225	2.65	4.55
SPPRS	65.6	11.4	0.3	5.8	1.8
Pooled with 95% CI	72.44 (95% CI 64.22, 80.65)	7.14 (95% CI 5.13, 9.15)	0.15 (95% CI 0.01, 0.28)	3.54 (95% CI 2.51, 4.56)	5.36 (95% CI 2.62, 8.10)

### The association between TB treatment outcome and HIV co-infection

**Fig. 5:**
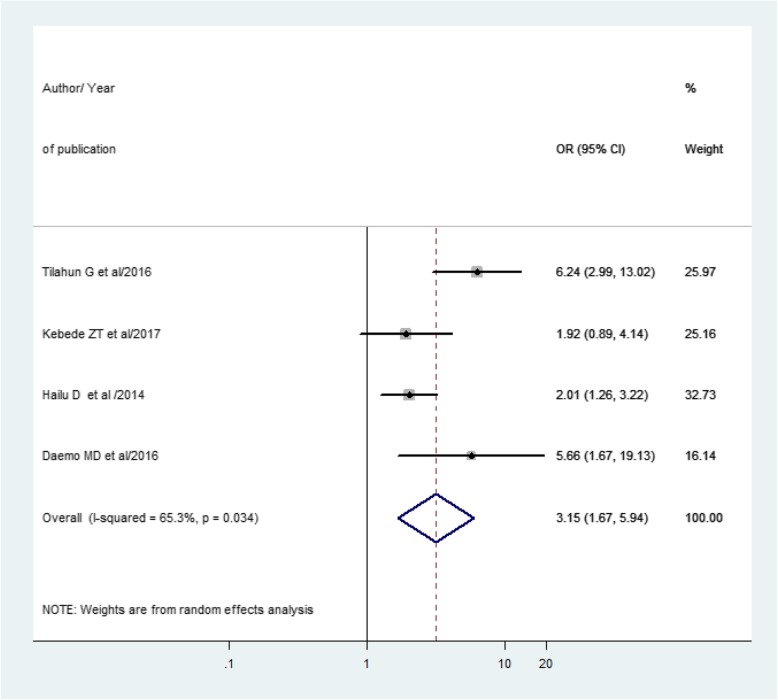
Forest plot that revealed the association between treatment outcome and HIV co-infection

To explore the association between TB treatment outcome and HIV co-infection, we used 4 studies that reported extractable data that helps to calculate the odds ratio of unsuccessful TB treatment among children with HIV co-infection. As illustrated in (Fig. 5), the overall pooled odds ratio of unsuccessful TB treatment outcome among HIV co-infection was 3.15 (95% CI 1.67, 5.94, *I*^2^ = 65.3%, *p* value = 0.034).

## Discussion

Tuberculosis is one of the global burdens that highly affect the vulnerable population like children. Successful treatment outcome of childhood tuberculosis is one of the mechanisms to reduce its transmission, drug resistance, mortality, and morbidity. This systemic review and meta-analysis had shown the treatment outcome of childhood tuberculosis and the impact of HIV on successful treatment outcomes in Ethiopia context. Despite a limited study regarding this topic in the country, we analyzed six studies to estimate the pooled treatment outcome of tuberculosis. In this systemic review, successful treatment outcome of childhood TB was 79.62% (95% CI 73.22, 86.02) which is in line with the study conducted in the Asia region (81.9%) [18]. However, it is lower than from the global recommendation suggested by the WHO which is ≥ 90% successful treatment outcome in the End TB strategy [1] and the study conducted from Bhutan Southeast Asia (93%) successful treatment outcome [19]. This might be due to the variations of the health service standards. In Asia, all children diagnosed with TB will be admitted for the first 2 months of treatment for directly observed therapy (DOT), whereas in Ethiopia no admission for directly observed therapy [20, 21].

A national surveillance study conducted in England and Wales reported that 88% of children completed their treatment which is higher than from our findings [22]. This might be due to the fact that in the developed countries, quality of care in all aspects is well improved which may have an impact in good treatment outcomes.

In Pakistan, one study showed that the overall successful treatment outcome (cured and treatment completed) was recorded as 95.1% which is higher than from our study [23].

The result of this study was congruent with a study conducted in Nigeria [24, 25]. In addition, it was in line with a study conducted from an international epidemiological database network with 80% of effective treatment outcomes among children taking anti-TB treatment [26].

On the other hand, the finding was higher than that of a study conducted in the Democratic Republic of Congo which was 70% successful treatment outcome and of this 69.6% was treatment completed [27].

### HIV/TB co-infection on children TB treatment outcome

In this systemic review and meta-analysis, 20.46% of children had poor treatment outcomes of which treatment failure was 0.15%, defaulter 5.36%, and death 3.5%. Consequently, poor TB treatment outcome was higher among children with HIV co-infection with an odds ratio of 3.15 (95% CI 1.67, 5.94) compared to HIV-negative children. One study from the international epidemiological database network also reported that HIV/TB children have poor treatment outcomes compared with HIV-non-infected children [26]. One of the major challenges in the management of children with HIV/TB co-infection is pharmacokinetics interaction of nevirapine-based ART regimen and rifampicin-based anti-tuberculosis treatment [28]. Another study which is conducted in Côte d’Ivoire on the impact of HIV infection on the outcome of tuberculosis among children revealed that children with HIV infection had a higher mortality rate than HIV negative [29]. The high death rate was reported from HIV-positive children which might be associated with immune-compromisation that results from other opportunistic infections like pneumonia, meningitis, and measles [30]. Unlike HIV sero-negative, TB/HIV-co-infected children would have the worst treatment outcome because they would have compromised immunity. As a result, they may not develop the signs and symptoms of infection unless it is detected through an advanced diagnostic modality [31]. The other reason for poor treatment outcome of TB/HIV co-infected children is the severity of illness. Hence, a serious illness is common in HIV-infected than HIV-negative children taking anti-tuberculosis treatment [32]. Therefore, a more severe illness which will end up with death is the most horrible outcome [10]. In this study, of the unfavorable treatment outcomes, defaulter is a paramount (5.36%), this might be associated with the high pill burdens especially in the case of children who took TB/HIV treatment and at a time had experienced intolerable side effect of drugs [33, 34], social stigma, having poor support from social/family, and the inaccessibility of the health care [21, 35, 36]. This poor treatment outcome might be due to poor adherence to the drugs and noncompliance to the health care services [36]. In such a condition, most patients shifted to traditional medicine, practicing cultural malpractice and doing various religious activities in the country [37]. This might be due to the nature of treatment that takes a long time to follow-up, patients may end in fatigue, and hopelessness on the medical treatment [31, 38].

### Limitation

Data was not found from some regions like Harari, Afar, Benshangul Gumze, Dire-Dawa, and Somali. Hence, the finding may not be representative of the aforementioned regions. Another limitation could be the likelihood of missing studies since not all databases were searched.

## Conclusion

The proportion of successful TB treatment outcomes in Ethiopia was found to be low compared to the threshold suggested by the WHO. HIV co-infection is significantly associated with poor treatment outcomes. Therefore, special attention is better to be given to children infected with both TB and HIV.

## Supplementary information

**Additional file 1.** The PRISMA guideline.

**Additional file 2.** The searching terms for PubMed and web of science.

**Additional file 3.** Searching terms used for Google Scholar.

**Additional file 4.** The JBI quality assessment tool for cohort and cross sectional studies.

## Data Availability

All data generated or analyzed during study are included in this systematic review and meta-analysis

## Abbreviations

DOT: Directly observed therapy
EPTB: Extra pulmonary tuberculosis
HIV: Human immunodeficiency virus
PTB: Pulmonary tuberculosis
SNNPRS: Southern Nations, Nationalities and Peoples Region State
TB: Tuberculosis
WHO: World Health Organization
