# Supplementary figures and images for: Childhood tuberculosis treatment outcome and its association with HIV co-infection in Ethiopia: a systematic review and meta-analysis
Source: Trop Med Health. 2020 Feb 18;48:7. doi: 10.1186/s41182-020-00195-x (PMC7027074; doi:10.1186/s41182-020-00195-x)

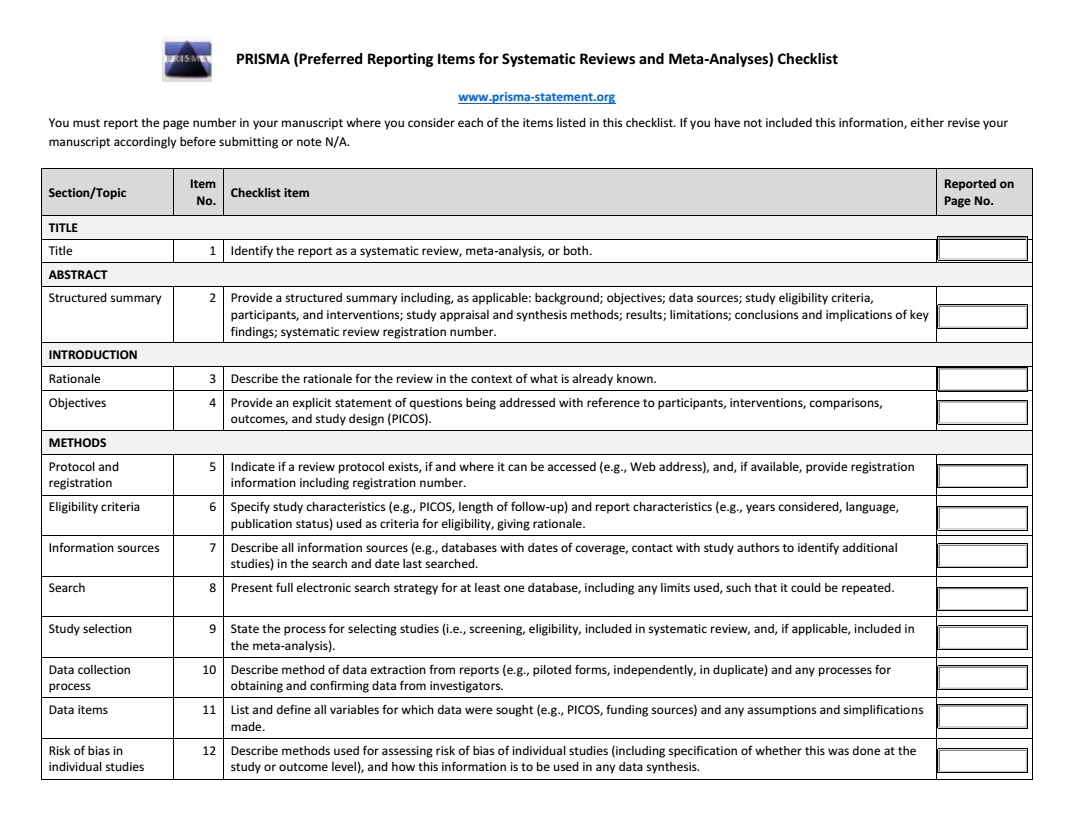


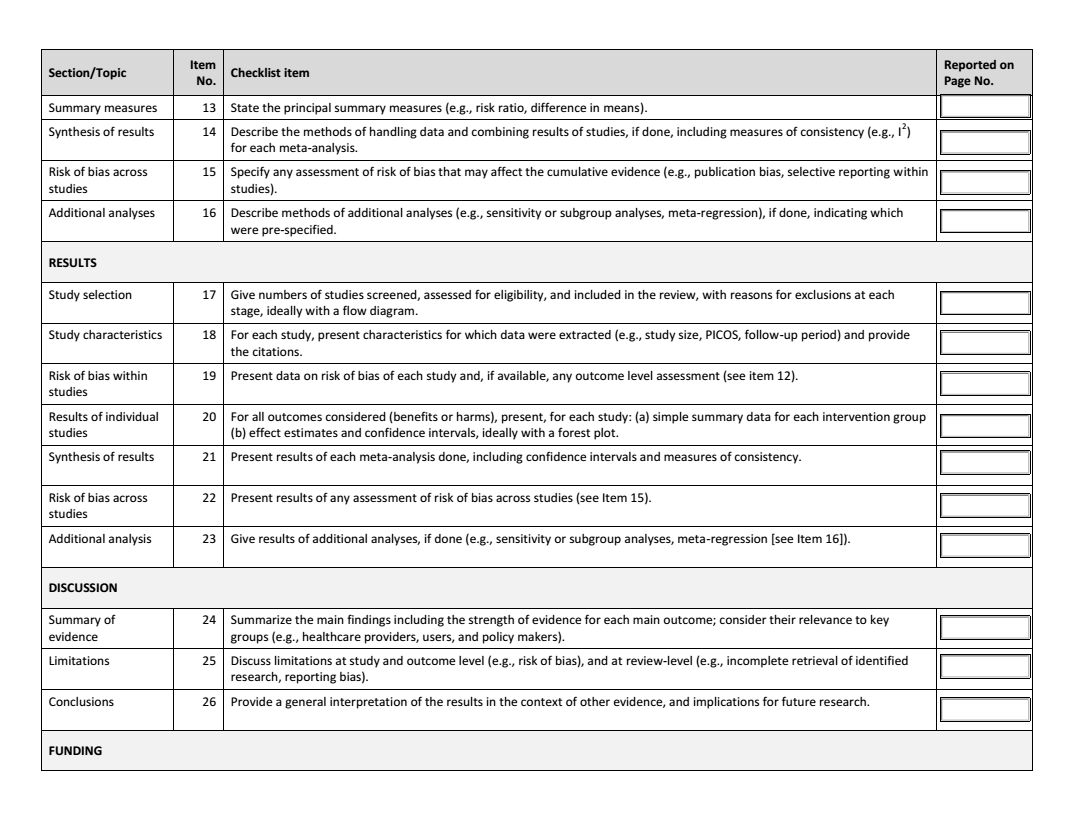

Supplement: Supplementary file 1 — Additional file 1. The PRISMA guideline. [file 41182_2020_195_MOESM1_ESM.docx]
