# Supplementary material for: Childhood tuberculosis treatment outcome and its association with HIV co-infection in Ethiopia: a systematic review and meta-analysis
Source: Trop Med Health. 2020 Feb 18;48:7. doi: 10.1186/s41182-020-00195-x (PMC7027074; doi:10.1186/s41182-020-00195-x)
Supplement: Supplementary file 2 — Additional file 2. The searching terms for PubMed and web of science. [file 41182_2020_195_MOESM2_ESM.docx]

((Tuberculosis OR TB [MeSH Terms] OR Mycobacterium Tuberculosis) AND (Treatment outcome OR successful treatment outcome [MeSH Terms] OR poor treatment outcome OR good treatment outcome [MeSH Terms] OR unsuccessful treatment) AND (children OR childhood [MeSH Terms] OR pediatrics OR less than 15 years children [MeSH Terms]) AND (HIV/co-infection OR TB/HIV co-infection [MeSH Terms] OR TB-HIV co-infection OR HIV infection [MeSH Terms]) AND (Ethiopia))
