# Supplementary material for: Childhood tuberculosis treatment outcome and its association with HIV co-infection in Ethiopia: a systematic review and meta-analysis
Source: Trop Med Health. 2020 Feb 18;48:7. doi: 10.1186/s41182-020-00195-x (PMC7027074; doi:10.1186/s41182-020-00195-x)
Supplement: Supplementary file 3 — Additional file 3. Searching terms used for Google Scholar. [file 41182_2020_195_MOESM3_ESM.docx]

((children TB treatment outcome OR childhood tuberculosis treatment outcome OR successful treatment outcome of tuberculosis among children) AND (HIV co-infection OR TB/HIV co-infection) AND (Ethiopia))
