# Supplementary material for: Childhood tuberculosis treatment outcome and its association with HIV co-infection in Ethiopia: a systematic review and meta-analysis
Source: Trop Med Health. 2020 Feb 18;48:7. doi: 10.1186/s41182-020-00195-x (PMC7027074; doi:10.1186/s41182-020-00195-x)

**JBI Critical Appraisal Checklist for Cohort Studies**

Reviewer Date

Author Year Record Number

|  | Yes | No | Unclear | Not applicable |
| --- | --- | --- | --- | --- |
| 1. Were the two groups similar and recruited from the same population? | □ | □ | □ | □ |
| 1. Were the exposures measured similarly to assign people   to both exposed and unexposed groups? | □ | □ | □ | □ |
| 1. Was the exposure measured in a valid and reliable way? | □ | □ | □ | □ |
| 1. Were confounding factors identified? | □ | □ | □ | □ |
| 1. Were strategies to deal with confounding factors stated? | □ | □ | □ | □ |
| 1. Were the groups/participants free of the outcome at the start of the study (or at the moment of exposure)? | □ | □ | □ | □ |
| 1. Were the outcomes measured in a valid and reliable way? | □ | □ | □ | □ |
| 1. Was the follow up time reported and sufficient to be long enough for outcomes to occur? | □ | □ | □ | □ |
| 1. Was follow up complete, and if not, were the reasons to loss to follow up described and explored? | □ | □ | □ | □ |
| 1. Were strategies to address incomplete follow up utilized? | □ | □ | □ | □ |
| 1. Was appropriate statistical analysis used? | □ | □ | □ | □ |

Overall appraisal: Include □ Exclude □ Seek further info □

Comments (Including reason for exclusion)


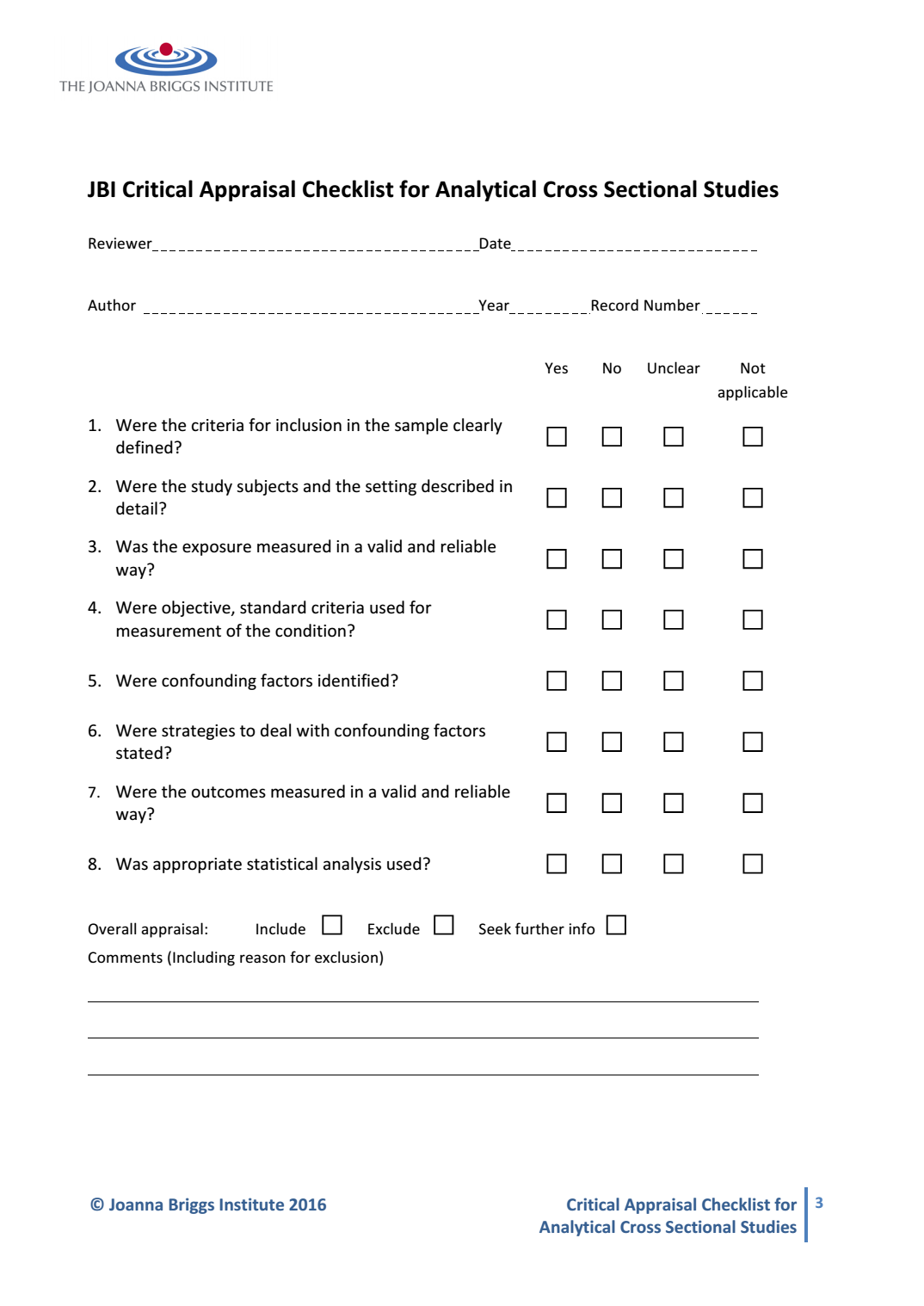

Supplement: Supplementary file 4 — Additional file 4. The JBI quality assessment tool for cohort and cross sectional studies. [file 41182_2020_195_MOESM4_ESM.docx]
